# Cu_7_S_4_ Nanozyme Efficiently Inactivating Drug‐Resistant Bacteria on Mouse Wound Models through Photodynamic and Photothermal Synergetic Therapy

**DOI:** 10.1002/advs.202503793

**Published:** 2025-06-09

**Authors:** Xueya Li, Dazhen Liu, Song Han, Yujia Liu, Yuanyang Fan, Yu Zhang, Chunchang Li, Zhenshan Xia, Lingbing Cui, Jing Cui, Jinghong Wen, Tao Yan, Chuanjia Jiang, Yongxin Jin, Qian Ren, Mingyang Liu

**Affiliations:** ^1^ State Key Laboratory of Medicinal Chemical Biology College of Life Nankai University Tianjin 300350 China; ^2^ Department of Urology Department of Neurology Department of Gynecology and Obstetrics Tianjin Medical University General Hospital Tianjin 300052 China; ^3^ College of Environmental Science and Engineering Nankai University Tianjin 300350 China; ^4^ Department of Anatomy and Embryology School of Basic Medical Sciences Suzhou Medical College of Soochow University Suzhou 215123 China; ^5^ School of Chemistry and Chemical Engineering Liaocheng University Liaocheng 252000 China

**Keywords:** Cu_7_S_4_ nanozyme, dual‐defect, efficient antibacterial, suitable electronic structure, synergetic process

## Abstract

The rapid emergence of drug‐resistant bacteria has outpaced the development of traditional antibiotics, necessitating the exploration of more effective therapeutic strategies. In this study, the design of a Cu_7_S_4_ multifunctional nanozyme, activated by near‐infrared (NIR) light is presented, that demonstrates enhanced antibacterial activity. Cu_7_S_4_ is synthesized with varying defect structures by utilizing different templates, which substantially optimize its absorption to H_2_O_2_ and lipopolysaccharides (LPS) molecules. This process generates an optimal electronic structure, producing efficient antibacterial activity through photodynamic and photothermal synergetic processes. Specifically, the Cu_7_S_4_ nanozyme with dual defects (*VCu* and *VCuCuCuSSS*) exhibits peroxidase‐like (POD), catalase‐like (CAT), and GSH‐depletion properties, effectively inactivating drug‐resistant bacteria such as Pseudomonas aeruginosa. Notably, in a mouse wound model infected with *P. aeruginosa*, the nanozyme demonstrates significant antibacterial efficacy, promoting wound healing under NIR light. This multifunctional Cu_7_S_4_ nanozyme presents a promising new strategy for combating drug‐resistant bacterial infections.

## Graphical abstract

Cu_7_S_4_ nanozymes, exhibiting peroxidase‐like, catalase‐like, and GSH‐depletion activities, are synthesized through defect engineering. These nanozymes demonstrate rapid inactivation of Gram‐negative bacteria, including *Escherichia coli* and *Pseudomonas aeruginosa*. In a mouse skin wound model infected with *P. aeruginosa*, the nanozyme showcases exceptional bactericidal effect.

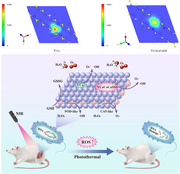

## 1 Introduction

Due to the diminishing supply of therapeutics targeting multidrug‐resistant (MDR) pathogens, drug‐resistant bacterial infections pose a pressing and escalating global public health crisis.^[^ ^1^ ^]^ Notably, the emergence of antibiotic‐resistant strains has rendered many infections untreatable, contributing to the deaths of 1.27 million people worldwide in 2019.^[^ ^2^ ^]^ Between 2019 and 2020, deaths from antimicrobial resistance (AMR) infections in the United States alone increased 15%, and it is projected that AMR will cause 10 million deaths annually by 2050.^[^ ^3^ ^]^ However, the discovery of new antibiotics has become increasingly challenging, with development costs exceeding $500 million and timelines extending over a decade.^[^ ^4^ ^]^ Alarmingly, the effective use period of new antibiotics is often less than three years due to the rapid emergence of resistance.^[^ ^5^ ^]^ Therefore, the urgent need for alternative therapeutic methods is evident.

Nanomaterials, with their unique properties and enzyme‐like catalytic activities, offer a promising alternative.^[^ ^6^ ^]^ For instance, peroxidase mimics can effectively decompose hydrogen peroxide (H_2_O_2_) into hydroxyl radicals (·OH),^[^ ^7^ ^]^ while catalase mimics decompose H_2_O_2_ into oxygen (O_2_), alleviating hypoxia and providing reaction substrates for photodynamic therapy.^[^ ^8^ ^]^ Bacterial infections typically create a proinflammatory microenvironment characterized by low pH, hypoxia, and abundant H_2_O_2_, providing an excellent response environment for the enzymatic activity of nanoenzymes.^[^ ^9^ ^]^ Furthermore, Glutathione (GSH) is an important antioxidant that removes free radicals under physiological conditions, protecting the sulfhydryl groups in the proteins and enzymes.^[^ ^10^ ^]^ Thus, multifunctional nanozymes with peroxidase‐like (POD), catalase‐like (CAT), and GSH‐depletion activities can generate abundant ROS to destroy biomolecules on which drug‐resistant bacteria depend.

Additionally, Antibacterial photodynamic therapy (aPDT), an environmentally friendly and efficient technology, has been extensively explored for inactivating drug‐resistant bacteria. It utilizes specific wavelengths of light to stimulate photosensitive nanomaterials to convert H_2_O and O_2_ into large amounts of toxic reactive oxygen species (ROS), which kill pathogenic microorganisms through oxidative damage to biomolecules (e.g., phospholipids, enzymes, proteins, and DNA).^[^ ^11^ ^]^ Due to its beneficial mechanism of action, aPDT exhibits broad‐spectrum performance and a low potential for resistance to pathogenic microorganisms. However, the effectiveness of this therapy depends critically on the properties of photocatalysts, which must penetrate the skin and reach infected tissues while ensuring the presence of sufficient O_2_. The activity of photocatalysts is influenced by their electronic structure,^[^ ^12^ ^]^ which depends on the band edge positions necessary for target chemical reactions.^[^ ^13^ ^]^ Defects in crystalline structures, such as vacancies, can significantly alter the mechanical, electrical, chemical, and thermal properties of nanomaterials.^[^ ^14^ ^]^ For example, sulfur vacancies in MoS_2_ nanosheets induce room‐temperature ferromagnetism,^[^ ^15^ ^]^ while oxygen vacancies in NiCo_2_O_4_@Pd nanozymes enhance catalytic activity.^[^ ^16^ ^]^ However, synthesizing photocatalysts with multifunctional nanozyme properties remains a significant challenge due to the complex interplay of composition, size, morphology, exposed crystal facets, and surface area.

Moreover, the antibacterial method of photothermal therapy (PTT) relies on the physical heat of nanomaterials to inactivate bacteria.^[^ ^17^ ^]^ According to Mie's theory, the plasmon band of spherical particles is attributed to the dipolar oscillations of the free electrons in the conduction band,^[^ ^18^ ^]^ apt electronic structure plays a pivotal role in the process. Nevertheless, although photothermal therapy to inactivate bacteria has drawn widespread attention, it remains an enormous challenge that PTT agents of design and synthesis take on favorable properties such as high photothermal conversion efficiencies, photostabilities, and biocompatibility to obtain effective inactivation. It is an ingenious strategy that a suitable electronic structure can modulate an apt photothermal process, leading to effective photodynamic and photothermal synergetic antibacterial.

In this study, we designed and synthesized two types of Cu_7_S_4_ nanoparticles via a solvothermal route, each possessing distinct dual‐defect structures: *VCu* and *VCuCuCuSSS* (Cu_7_S_4_‐1) or *VCu* and *VCuCuCuCuCuS* (Cu_7_S_4_‐2). The presence of these defects gives the Cu_7_S_4_ nanoparticles peroxidase (POD), catalase (CAT), and glutathione (GSH) depletion activities as well as excellent photocatalytic performance, which not only generates a large amount of ROS to induce bacterial oxidative damage but also generates O_2_ as a substrate for its photocatalysis, which makes up for the defect that general photocatalysts are limited by low oxygen content. Meanwhile, density‐functional theory (DFT) calculations and experimental results showed that Cu_7_S_4_‐1 has a better uptake capacity for H_2_O_2_, especially lipopolysaccharide (LPS), compared with Cu_7_S_4_‐2, which is significantly different from other reported nanozymes. In addition, Cu_7_S_4_‐1 possessed photodynamic and photothermal synergistic effects, which enabled effective inactivation of drug‐resistant bacteria such as *E. coli* and *P. aeruginosa*. Furthermore, in vivo antibacterial assays using a mouse wound model infected with *P. aeruginosa* confirmed that Cu_7_S_4_‐1, assisted by NIR light, possesses exceptional antibacterial efficacy, providing a promising strategy for combating MDR bacterial infections.

## 2 Results and Discussion

### 2.1 Characterization of Cu_7_S_4_

**Figure 1:**
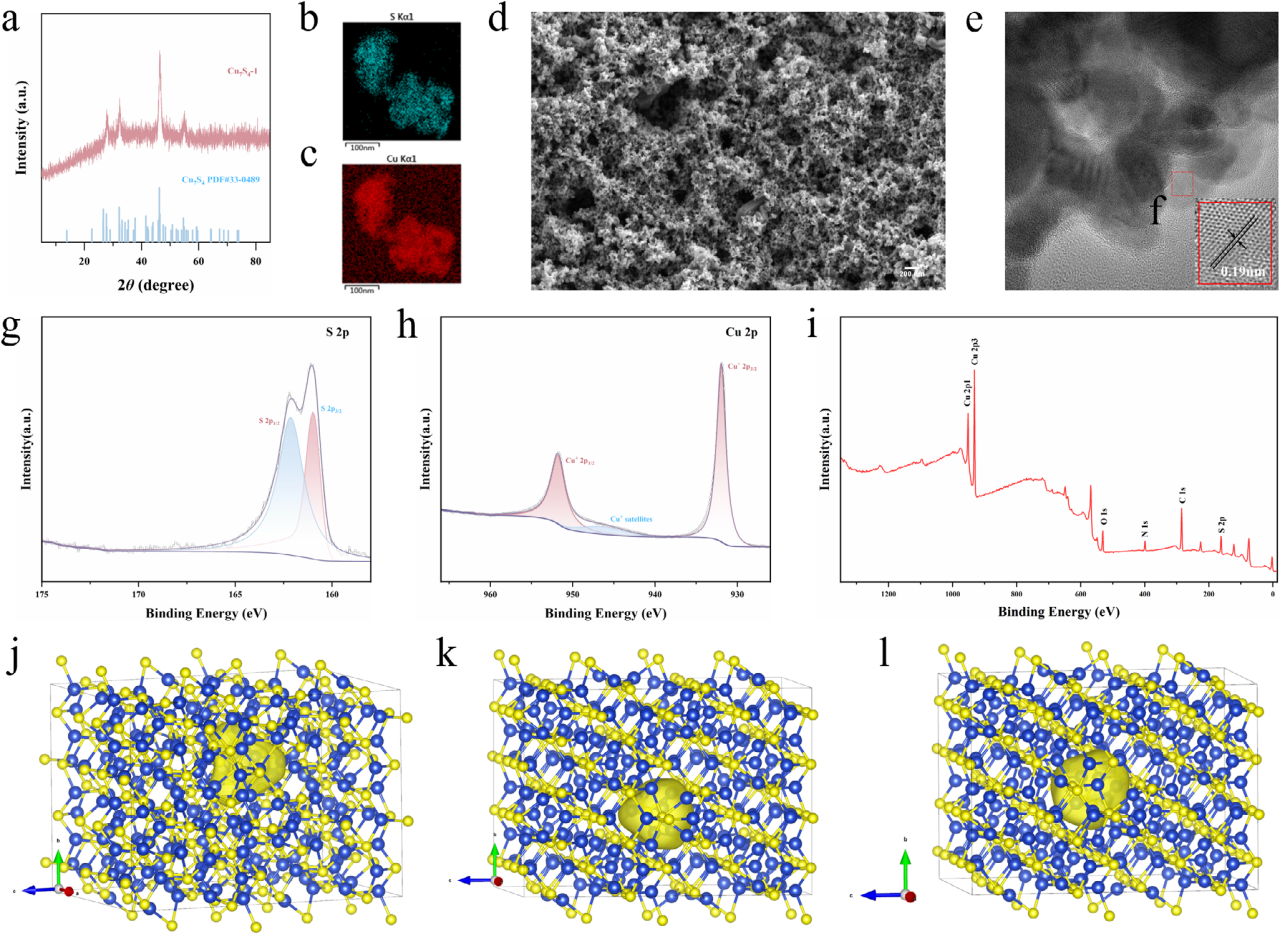
a) XRD pattern of Cu_7_S_4_‐1. b, c) mapping images of Cu_7_S_4_‐1. b) show S element. c) show Cu element, d) SEM images of Cu_7_S_4_‐1. e) TEM image of Cu_7_S_4_‐1. (f) lattice spacings of the image of Cu_7_S_4_‐1. g–i) XPS images of Cu_7_S_4_‐1. j) defect *VCu* image of Cu_7_S_4_‐1. k) defect *VCuCuCuSSS* image of Cu_7_S_4_‐1. l) defect *VCuCuCuCuCuS* image of Cu_7_S_4_‐2.

The two synthesized Cu_7_S_4_ samples were thoroughly characterized. X‐ray diffraction (XRD) patterns revealed that the Cu_7_S_4_ nanoparticles predominantly grew along the (224) facet (**Figure** **1**a; Figure S1a, Supporting Information), exhibiting a well‐indexed orthorhombic phase (Pnma (62)) with lattice parameters: a = 7.0965 Å, b = 7.8223Å, c = 11.078 Å (JCPDS No. 33‐0489). The absence of impurities or additional phase peaks in the XRD patterns confirmed the purity of the samples. The elemental mapping demonstrating the presence and uniform distribution of Cu and S across the nanoparticle surfaces (Figure 1b,c; Figure S1b,c,d, Supporting Information). Low‐magnification scanning electron microscopy (SEM) image revealed a characteristic small 2D morphology (Figure 1d; Figure S1e, Supporting Information), which was corroborated by transmission electron microscopy (TEM) image (Figure 1e; Figure S1f, Supporting Information), showing particle diameters of ≈30–50 nm. High‐resolution TEM (HRTEM) images displayed lattice spacings of 0.19 nm (Figure 1f), consistent with the XRD analysis.

X‐ray photoelectron spectroscopy (XPS) was employed to analyze the elemental composition and surface chemical states of the Cu_7_S_4_ sample. The high‐resolution XPS spectra for Cu_7_S_4_‐1 (Figure 1g,h,i) showed Cu 2p_1/2_ and Cu 2p_3/2_ binding energy peaks at 951.9 and 931.9 eV, respectively. For both samples, S 2p_1/2_ and S 2p_3/2_ peaks appeared at 162.3 and 161.2 eV, respectively. Notably, the sample exhibited shifts to lower energies,^[^ ^19^ ^]^ attributable to electron enrichment caused by the presence of defects.^[^ ^20^ ^]^

Positron annihilation spectroscopy was used to characterize defects within the Cu_7_S_4_ samples. The results indicated four‐lifetime components (Tables S1 and S2, Supporting Information) for each sample. The two longest components (τ_3_ and τ_4_) were associated with positron annihilation in large defect clusters or interfaces.^[^ ^21^ ^]^ The shorter components for Cu_7_S_4_‐1 (τ_1_ = 221.6 ps, τ_2_ = 355.0 ps) were attributed to the *VCu* and *VCuCuCuSSS* defect (Figure 1j,k), with relative intensities of 29.1% and 67.5%, respectively.^[^ ^22^ ^]^ Similarly, for Cu_7_S_4_‐2, the shorter components (τ_1_ = 226.0 ps, τ_2_ = 374.0 ps) corresponded to *VCu* and *VCuCuCuCuCuS* defects (Figure 1l), with relative intensities of 34.5% and 59.6%, respectively. Both Cu_7_S_4_ samples exhibited dual defects.

**Figure 2:**
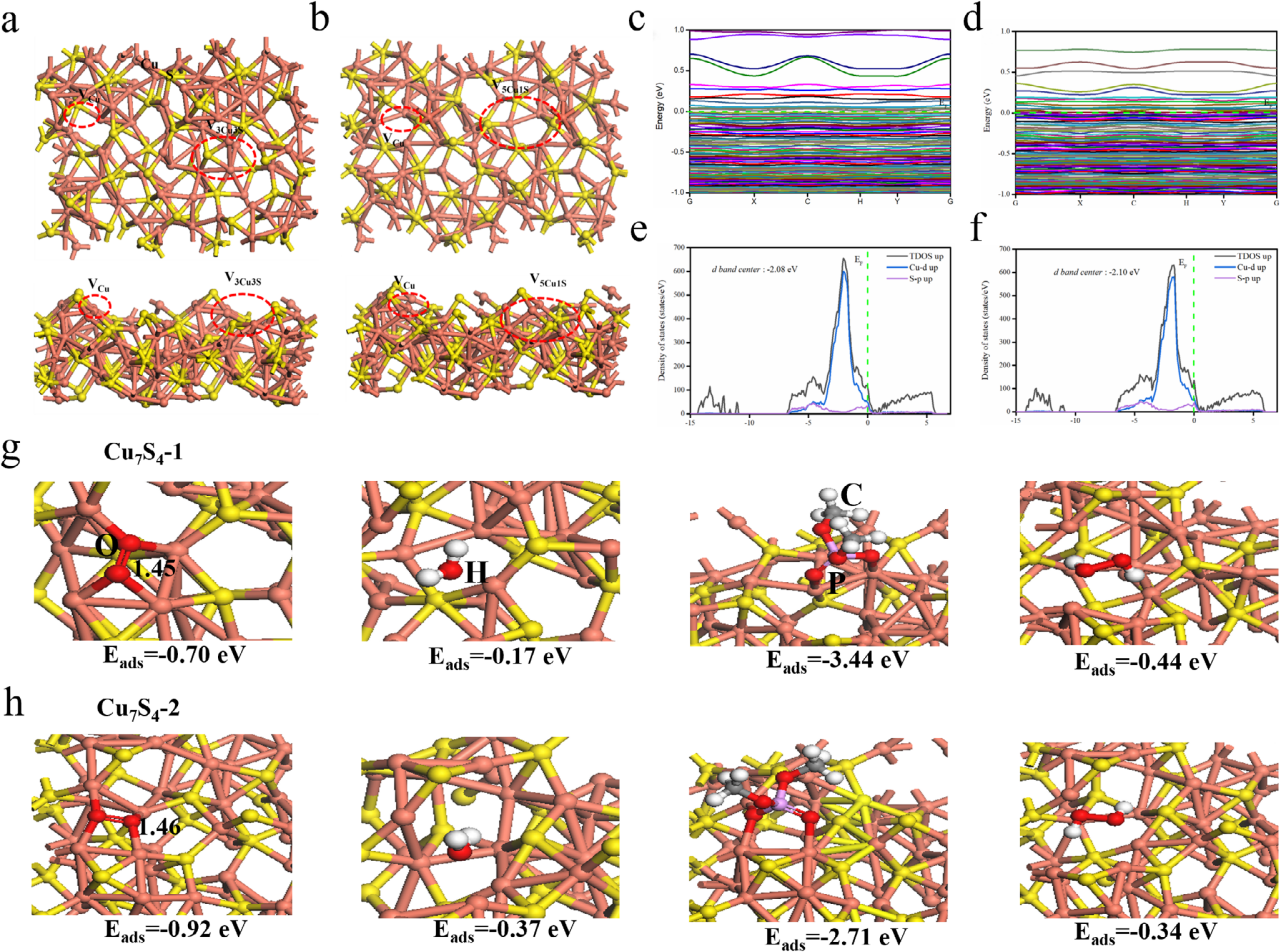
a) the optimized geometries of Cu_7_S_4_‐1. b) the optimized geometries of Cu_7_S_4_‐2. c) the band structure and e) DOS of Cu_7_S_4_‐1. d) the band structure and f) DOS of Cu_7_S_4_‐2. g) the optimized geometries of O_2_, H_2_O, LPS and H_2_O_2_ adsorbed on Cu_7_S_4_‐1. h) the optimized geometries of O_2_, H_2_O, LPS and H_2_O_2_ adsorbed on Cu_7_S_4_‐2.

To investigate the influence of defects on the physicochemical properties of Cu_7_S_4_, we employed first‐principles calculations. Based on the intact Cu_7_S_4_ (224), we constructed two structures with different defect species to compare their bactericidal capabilities, namely Cu_7_S_4_‐1 (contains *V_Cu_* and *V_3Cu3S_*) and Cu_7_S_4_‐2 (contains *V_Cu_* and *V_5Cu1S_*), which shown in **Figure** **2**a,b.

The effect of promotion is assessed via the comparison of adsorption energies, calculated as:

(1) Eads=E(adsorbate/slab)−E(slab)−E(adsorbate)

Where *E_(adsorbate/slab)_* is the energy of the slab with bound adsorbate, *E_(slab)_* is the energy of the fully relaxed slab, and *E_(adsorbate)_* is the energy of the free adsorbate placed in an empty cell of the same dimensions as that used to define the slab model.

The band structure and density of states (DOS) were further analyzed to study the electronic properties of the two catalysts. For Cu_7_S_4_‐1, the band gap was only 0.02 eV (Figure 2c) and for Cu_7_S_4_‐2, the band gap was further reduced (Figure 2d). From Figure 2d, the conduction band and valence band were crossing at the Fermi level, resulting in a band gap of almost 0 eV. For the plot of DOS (Figure 2e,f), the Fermi level is mainly contributed by the d orbital of Cu and the p orbital of S. Moreover, the d band center of Cu_7_S_4_‐1 is −2.08 eV which was closer to the Fermi level than that of Cu_7_S_4_‐2 (−2.10 eV). Therefore, it can be speculated that Cu_7_S_4_‐1 has better catalytic performance.

The O_2_ molecules were bound to the Cu atoms through two O atoms with the O─O bond lengths were 1.45 and 1.46 Å, respectively. The adsorption energy for O_2_ adsorbed on Cu_7_S_4_‐1 and Cu_7_S_4_‐2 were −0.70 and −0.92 eV. The H_2_O was physically adsorbed upon the defects with the adsorption energy of −0.17 and −0.37 eV, respectively. The LPS was binding to the Cu atoms through two O atoms with the adsorption energy of −3.44 and −2.71 eV. H_2_O_2_ adsorbed upon the defects with the O─O bond lengths were 1.47 and 1.48 Å, respectively. And the adsorption energy of H_2_O_2_ was −0.44 and −0.34 eV (Figure 2g,h). The adsorption results indicated that while Cu_7_S_4_‐2 demonstrated effective adsorption of H_2_O and O_2_, Cu_7_S_4_‐1 exhibited superior adsorption of Lipopolysaccharide (LPS), a component of the cell wall of Gram‐negative bacteria.^[^ ^23^ ^]^ The presence of LPS provided an effective anchoring site for the nanozymes, thereby enhancing their efficacy against bacteria. Furthermore, compared to Cu_7_S_4_‐2, Cu_7_S_4_‐1 demonstrated a higher capacity to adsorb H_2_O_2_, providing more substrates for its POD‐like activity. This enhanced generating capacity of ROS was consistent with the experimental results.

**Figure 3:**
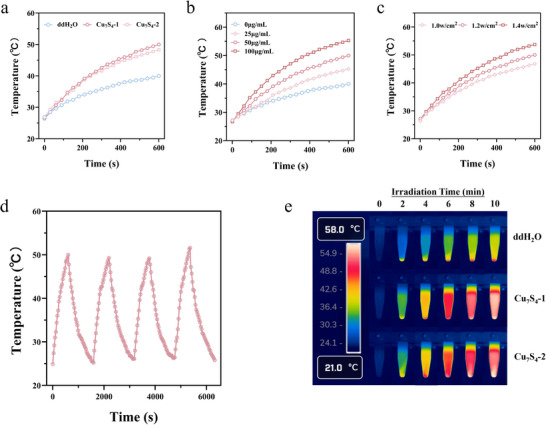
a) Temperature curves of Cu_7_S_4_‐1, Cu_7_S_4_‐2 aqueous solution (50 µg mL^−1^) and ddH_2_O under 808 nm laser (1.2 W cm^−2^). b) Temperature curves of different concentration of Cu_7_S_4_‐1 (0, 25, 50, and 100 µg mL^−1^) under 808 nm laser (1.2 W cm^−2^). c) Temperature curves of Cu_7_S_4_‐1 aqueous solution (50 µg mL^−1^) under different power densities of 808 nm laser irradiation (1.0, 1.2, and 1.4 W cm^−2^). d) Heating and cooling curves of Cu_7_S_4_‐1 aqueous solution (50 µg mL^−1^) under irradiation of 808 nm laser (1.2 W cm^−2^). (e) Infrared thermal images of Cu_7_S_4_ aqueous solution (50 µg mL^−1^) under 808 nm laser irradiation (1.2 W cm^−2^).

As Cu_7_S_4_‐1 also exhibits excellent optical absorption properties in the first NIR‐I region (700–900 nm), its photothermal conversion properties under 808 nm laser irradiation were further investigated. As shown in **Figure** **3**, the photothermal properties of Cu_7_S_4_‐1 aqueous solution under 808 nm laser illumination show an obvious enhancement trend with the increase of its concentration and the laser power density. In addition, the four cycles of heating and cooling curves indicate that Cu_7_S_4_‐1 has good photothermal stability in comparison with Cu_7_S_4_‐2. Therefore, the above results indicate that Cu_7_S_4_‐1 has the potential to be used as a new type of photothermal therapy (PTT) agent.

### 2.2 Antibacterial Performance of Cu_7_S_4_ in *E. coli*

**Figure 4:**
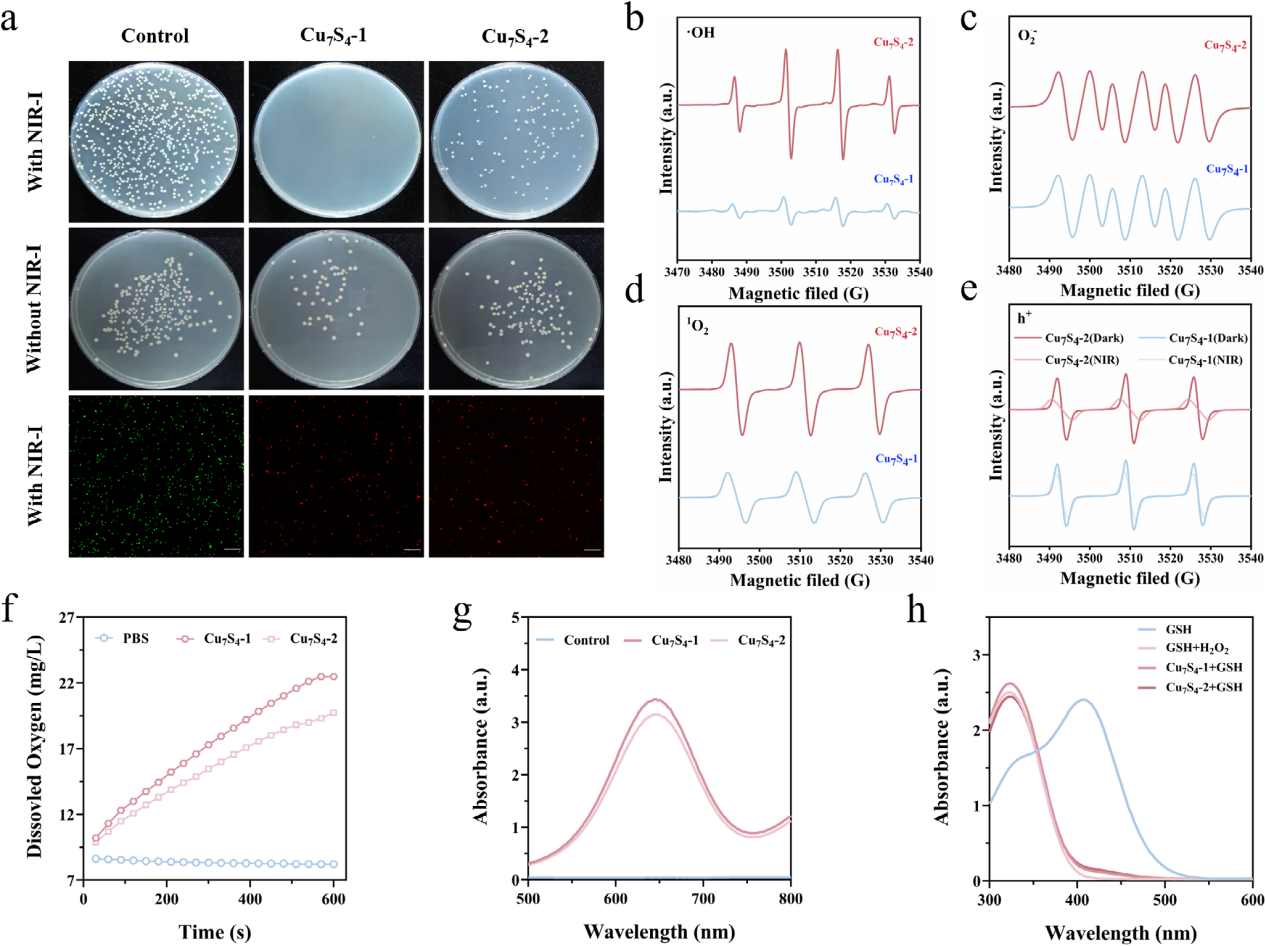
a) Antibacterial effects of Cu_7_S_4_ on *E. coli*, Colony of *E. coli* exposed with Cu_7_S_4_‐1 and Cu_7_S_4_‐2, with or without 808 nm laser irradiation (1.2 W cm^−2^) for 10 min and Live/dead fluorescence images of *E. coli*, scale bar, 20 µm. ESR spectra detection of b) ·OH, c) O_2·_^−^, d) ^1^O_2_, e) h^+^. f) CAT‐like activities of Cu_7_S_4_ for O_2_ generation. The low amount of O_2_ at 0 s is attributed to the dissolved O_2_ in water. g) UV–vis absorption of TMB catalyzed by Cu_7_S_4_ in the presence of air, H_2_O_2_. h) absorption spectra of consumed GSH after treatment with Cu_7_S_4_.

The plate counting test was utilized to evaluate the antibacterial activity of Cu_7_S_4_. Two types of Cu_7_S_4_ nanoparticles, each at a concentration of 50 µg mL^−1^, were tested against *E. coli* for 30 min. A control group without Cu_7_S_4_ was also included. Without NIR‐I (808 nm) irradiation, Cu_7_S_4_‐1 treatment caused an 80.42% killing effect, while Cu_7_S_4_‐2 treatment only caused 53.38%. However, under NIR‐I (808 nm) irradiation, the bacteria's functionality was significantly impaired. Experimental results demonstrated that Cu_7_S_4_‐1 achieved 99.99% *E. coli* inactivation, while Cu_7_S_4_‐2 inactivated only 85.13% of *E. coli*, as shown in **Figure** **4**a. To further investigate the antibacterial effects on *E. coli*, Confocal fluorescence microscopy was employed to examine cell membrane disruption. Using SYTO9 dye, live bacteria emitted green fluorescence, whereas dead bacteria emitted red fluorescence after propidium iodide (PI) staining. The results revealed that nearly all bacterial cell membranes were damaged following incubation with Cu_7_S_4_‐1 under NIR‐I (808 nm) irradiation. In contrast, only partial membrane disruption was observed in *E. coli* incubated with Cu_7_S_4_‐2 under the same conditions, as illustrated in Figure 4a. Our investigation revealed that Cu_7_S_4_‐1 possessed higher photothermal conversion and temperature increased higher than that of Cu_7_S_4_‐2 and exhibited more efficient antibacterial activity through the photodynamic and photothermal synergetic process under NIR‐I (808 nm) irradiation, although Cu_7_S_4_‐2 can form more ROS under the light.

To further explore the antibacterial mechanism and differences between the two Cu_7_S_4_ samples, electron spin resonance (ESR) experiments were conducted to detect various reactive oxygen species (ROS), including hydroxyl radicals (·OH) (Figure 4b), superoxide anions (O_2_^·−^) (Figure 4c), singlet oxygen (^1^O_2_) (Figure 4d), and holes (h^+^) (Figure 4e) under NIR irradiation. Notably, Cu_7_S_4_‐2 produced higher levels of ·OH and h^+^ compared to Cu_7_S_4_‐1. However, as previously stated, Cu_7_S_4_‐1 demonstrated more pronounced adsorption of LPS and H₂O₂. Consequently, Cu_7_S_4_‐1 exhibited a more pronounced bactericidal effect, as evidenced by the bacterial plate experiments and the measurements of enzyme‐like activities.

CAT‐like activity was assessed by measuring the increase in dissolved oxygen concentration in H_2_O_2_‐treated solutions. Cu_7_S_4_‐1 demonstrated higher oxygen generation compared to Cu_7_S_4_‐2 (Figure 4f), indicating that Cu_7_S_4_‐1 has stronger CAT‐like activity, facilitating the decomposition of H_2_O_2_ into O_2_ molecules in the proinflammatory microenvironment of bacterial infection, thereby alleviating hypoxia. POD‐mimicking activity was characterized by monitoring the absorbance change at 652 nm, which corresponds to the efficient oxidation of 3,3′,5,5′‐tetramethylbenzidine (TMB) in an oxygen system. The results showed that Cu_7_S_4_‐1 exhibited superior POD‐like properties compared to Cu_7_S_4_‐2 (Figure 4g), suggesting that Cu_7_S_4_‐1 more efficiently decomposes H_2_O_2_ into ·OH in the proinflammatory microenvironment of bacterial infections. Furthermore, we investigated its GSH‐consuming ability through Ellman's assay.^[^ ^24^ ^]^ The characteristic peaks of DTNB (5, 5′‐dithiobis (2‐nitrobenzoic acid)) at 412 nm were decreased significantly within 60 min post‐treatment with Cu_7_S_4_ (Figure 4h).

### 2.3 Transcriptomics Analysis of *E. coli* Treated with Cu_7_S_4_ Nanomaterials

**Figure 5:**
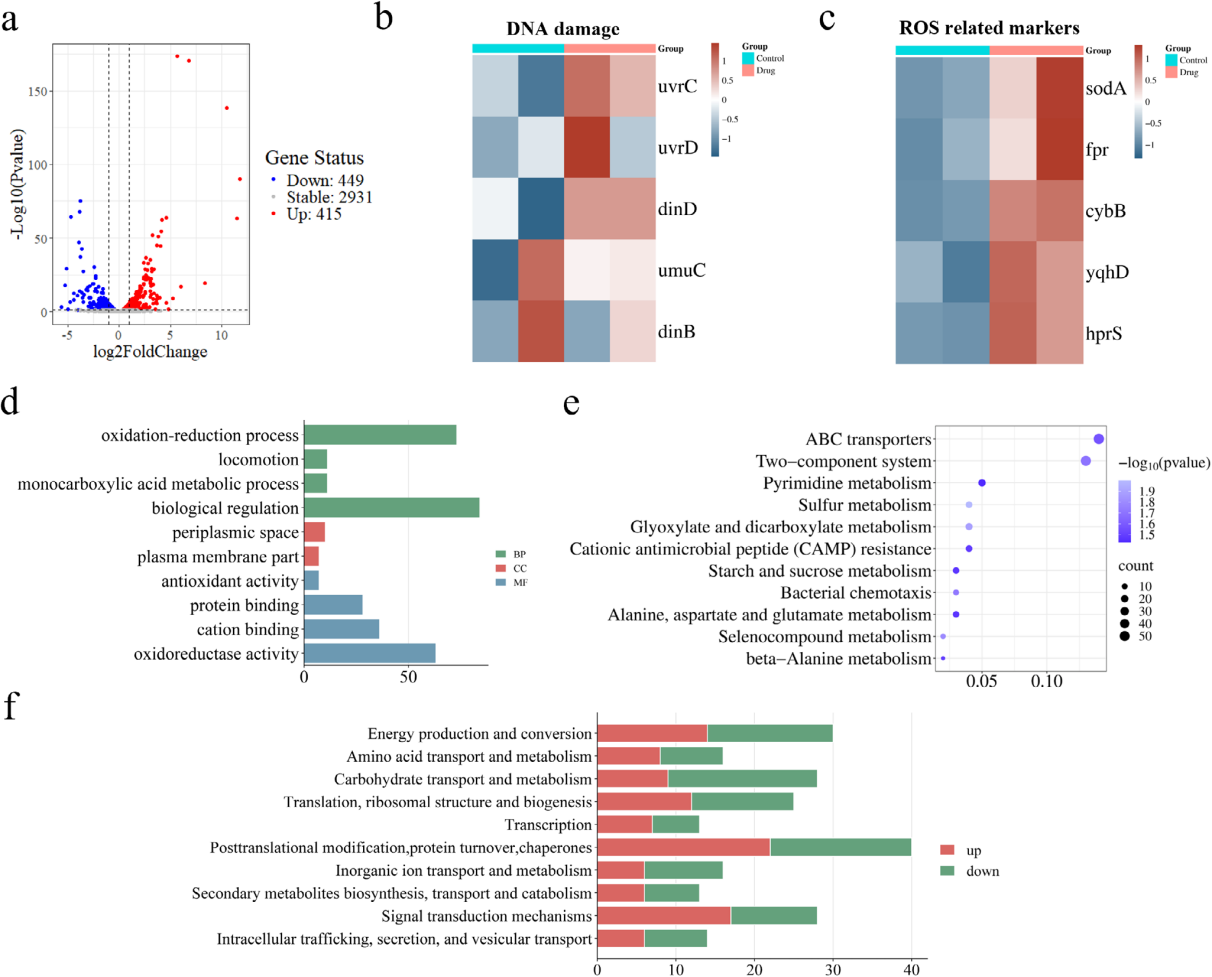
Transcriptomics analysis of *E. coli* treated with Cu_7_S_4_‐1 nanomaterials. a) The volcano map displays DEGs between Cu_7_S_4_‐1 nanomaterials and control groups. b) The heatmap showing DEGs of DNA damage between Cu_7_S_4_‐1 nanomaterials and control groups. c) The heatmap showing DEGs of ROS‐related markers between Cu_7_S_4_‐1 nanomaterials and control groups. d) GO enrichment analysis of DEGs between Cu_7_S_4_‐1 nanomaterials and control groups. e) KEGG enrichment analysis of different pathways in Cu_7_S_4_‐1 nanomaterials compared to control in accordance with DEGs. f) COG classifications of the DEG set between Cu_7_S_4_‐1 nanomaterials and control groups.

To systematically elucidate the mechanism by which Cu_7_S_4_‐1 nanomaterials affect *E. coli*, transcriptome RNA‐seq was performed. A volcano plot displayed the differentially expressed genes (DEGs) between the Cu_7_S_4_‐1‐treated and control groups, revealing 415 upregulated and 449 downregulated genes following a 5‐min exposure to Cu_7_S_4_ nanomaterials (**Figure** **5**a). Notably, DNA damage and repair genes such as dinD and uvrC showed significant upregulation compared to the control group (Figure 5b). It has been posited that the presence of Cu_7_S_4_ nanozymes generates a substantial quantity of ROS, which in turn causes damage to bacterial DNA, ultimately leading to its demise. Additionally, oxidative stress‐related genes, including sodA and hprS, were also upregulated (Figure 5c), further demonstrating that ROS production induces oxidative stress and damage in bacteria. Gene Ontology (GO) analysis indicated that exposure to Cu_7_S_4_‐1 nanomaterials led to specific enrichment of DEGs in biological processes such as oxidation‐reduction, locomotion, monocarboxylic acid metabolism, and biological regulation. Enrichment was also observed in cellular components like the periplasmic space and plasma membrane, as well as in molecular functions including protein binding, cation binding, and oxidoreductase activity, compared with the control group (Figure 5d), which was hypothesized to be possibly due to the disruption of biological macromolecules such as proteins, phospholipids and metabolism of the bacteria by the ROS‐induced oxidative stress. KEGG pathway enrichment analysis further revealed significant differences in sulfur metabolism, glyoxylate and dicarboxylate metabolism, bacterial chemotaxis, and selenocompound metabolism in the Cu_7_S_4_‐1‐treated group (Figure 5e). Finally, COG classifications of DEGs showed that the Cu_7_S_4_‐1‐treated group had more upregulated DEGs related to post‐translational modification, protein turnover, chaperones, signal transduction mechanisms, and transcription, while downregulated DEGs were enriched in carbohydrate transport and metabolism, as well as inorganic ion transport and metabolism (Figure 5f).

### 2.4 Antibacterial Performance of Cu_7_S_4_ in *P. aeruginosa*

**Figure 6:**
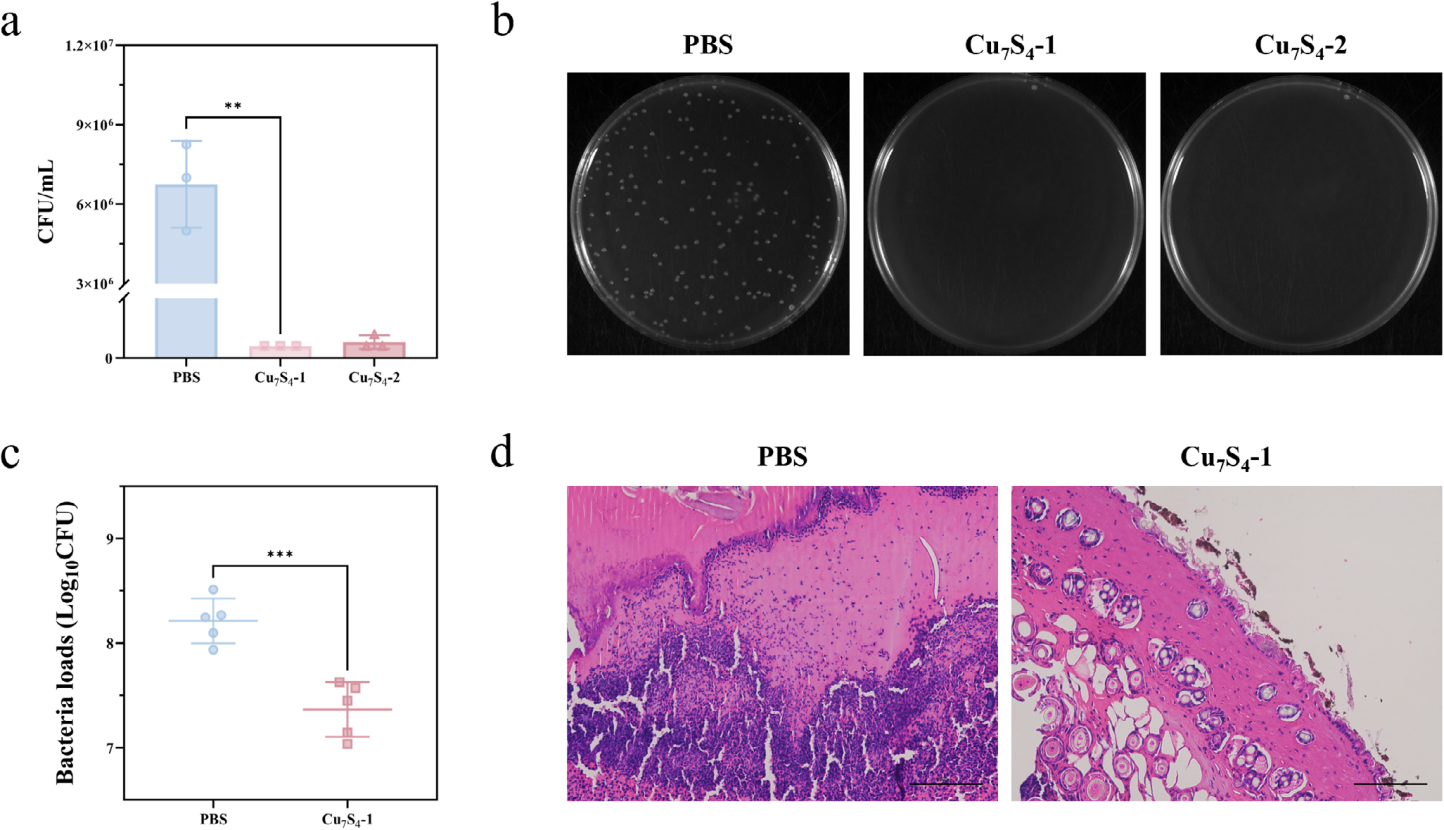
a) Colony forming unit (CFU) counting assay of bacteria treated with PBS, Cu_7_S_4_‐1 nanozymes, and Cu_7_S_4_‐2 nanozymes. (*n* = 3 independent experiments). b) Plate counting test of bacteria treated with nanozymes. c) Bacteria loading in the wound tissue of control mice and mice treated with Cu_7_S_4_ on day 6. *n* = 5 mice per group and d) HE‐stained images of wounds in the control group and Cu_7_S_4_‐1 group at 6 days of treatment, scale bar, 100 µm. ^**^ indicated a significant difference between the group exposed to irradiation (*p* < 0.01), ^***^ indicated a significant difference between the group exposed to irradiation (*p* < 0.001).

To further investigate the bacterial killing activity of the Cu_7_S_4_ nanozyme in vitro, we examined its ability to inactivate *P. aeruginosa* using the colony counting method. Compared to Cu_7_S_4_‐2, Cu_7_S_4_‐1 nanozyme demonstrated superior antibacterial activity at a concentration of 50 µg mL^−1^ (**Figure** **6**a,b). Additionally, an in vivo antibacterial model was established using mouse skin wounds infected with the *P. aeruginosa* standard strain PAO1. The infected wounds were treated with Cu_7_S_4_‐1 nanozyme and subjected to NIR irradiation. On Day 6 post‐treatment, bacterial burden and histopathological observations were assessed. As shown in Figure 6c, wounds treated with Cu_7_S_4_‐1 nanozyme exhibited significantly lower bacterial loads compared to the PBS control group. Correspondingly, hematoxylin and eosin (HE) staining images (Figure 6d) revealed a pronounced accumulation of immune cells in the control group (treated with PBS), indicating severe inflammation at the wound sites. In contrast, wounds treated with Cu_7_S_4_‐1 nanozyme showed a marked reduction in immune cell infiltration. These findings demonstrate that Cu_7_S_4_ nanozyme possesses substantial anti‐inflammatory properties, likely attributable to its high antibacterial efficiency in treating *P. aeruginosa*‐infected wounds.

## 3 Conclusion

In summary, we designed and synthesized two Cu_7_S_4_ nanozymes through defect engineering to inactivate drug‐resistant bacteria. By employing two types of template‐controlled synthetic processes, Cu_7_S_4_ with distinct dual‐defect configurations was formed. Experimental results and density functional theory (DFT) computations revealed that the electronic structures of the Cu_7_S_4_ nanozymes were effectively tuned, imparting them with multifunctional enzyme‐like properties, including peroxidase (POD)‐like, catalase (CAT)‐like, and glutathione (GSH)‐depletion activities. Compared to Cu_7_S_4_‐2 with dual defects of *VCu* and *VCuCuCuCuCuS*, the Cu_7_S_4_‐1 nanozyme with dual defects of *VCu* and *VCuCuCuSSS* exhibited superior adsorption properties for H_2_O_2_ molecules, and lipopolysaccharides (LPS) and apt electronic structure, leading to the formation of suitable ROS and photothermal temperature, exhibited synergistically efficient antibacterial activity against drug‐resistant bacteria. Notably, the Cu_7_S_4_ nanozyme with dual defects *VCu* and *VCuCuCuSSS* effectively treated *P. aeruginosa*‐infected wounds in vivo under NIR irradiation, as demonstrated in a mouse wound model. Our research provides valuable insights into the design of novel nanozymes with efficient antibacterial properties.

## Conflict of Interest

The authors declare no conflict of interest.

## Supporting information



Supporting Information

## Data Availability

The data that support the findings of this study are available in the supplementary material of this article.
