# Supplementary material for: Cu7S4 Nanozyme Efficiently Inactivating Drug‐Resistant Bacteria on Mouse Wound Models through Photodynamic and Photothermal Synergetic Therapy
Source: Adv Sci (Weinh). 2025 Jun 9;12(33):e03793. doi: 10.1002/advs.202503793 (PMC12412585; doi:10.1002/advs.202503793)
Supplement: Supplementary file 1 — Supporting Information [file ADVS-12-e03793-s001.docx]

Supporting Information

**Cu_7_S_4_ nanozyme efficiently Inactivating Drug-Resistant Bacteria on Mouse Wound Model through Photodynamic and Thermodynamic Synergetic Therapy**

Xueya Li^#^, Dazhen Liu^#^, Song Han^#^, Yujia Liu, Yuanyang Fan, Yu Zhang, Chunchang Li, Zhenshan Xia, Lingbing Cui, Jing Cui, Jinghong Wen*, Tao Yan, Chuanjia Jiang, Yongxin Jin*, Qian Ren* and Mingyang Liu*

^#^These authors contributed equally to this work.

**Experimental Section**

**1.1. Synthesis of Cu_7_S_4_ nanozymes**

Two-step approach to synthesis is used in this process. 0.3g of CuAc_2_ was added into a 50 mL beaker, 0.4g of surfactant PVP8000 (or PVP40000) was added, and then 40 ml of *N, N*-dimethylacetamide and 200 μL of aniline were added with magnetic stirring for 0.5 hours to form a homogeneous solution, then 0.15g of thiourea was added. Subsequently, the mixture was transferred into a Teflon-lined stainless steel autoclave with a capacity of 50 ml for solvothermal treatment at 150 °C for 20 h. After the autoclave was allowed to cool to room temperature, the precipitate was separated by centrifugation, then washed with water and absolute ethanol to remove impurities, and the Cu_7_S_4_ nanozymes were obtained.

**1.2.** **Characterization**

The phase of the samples was characterized by X-ray diffraction (XRD) under a Rigaku D/Max (EAST) Ultima II Powder XRD 6s X-ray diffractometer employing Cu Kα radiation, λ = 1.54056 Å. The morphology and size of the samples were characterized by using a field emission scanning electron microscope (FEI Quanta 400 ESEM FEG) and transmission electron microscopy (JEOL 2100 Field Emission Gun Transmission Electron Microscope). Electrochemical impedance spectroscopy (EIS) was performed on dummy cells with a typical symmetric sandwich-like structure, i.e., CE/electrolyte/CE, using an impedance measurement unit of the workstation (electrochemical workstation, CS310) in the frequency range, 0.1–10^6^ Hz, with an ac amplitude of 10 mV.

**1.3. Positron Annihilation Measurement**

The positron lifetime experiments were carried out with a fast-slow coincidence ORTEC system with a time resolution of ~230 ps full width at half-maximum. A 5mCi source of ^22^Na was sandwiched between two identical samples, and the total count was 1 million. Positron lifetime calculations were performed using the ATSUP method,^[1]^ in which the electron density and the positron crystalline Coulomb potential are constructed by the non-self-consistent superposition of free atom electron density and Coulomb potential in the absence of the positron. Our calculations of the positron lifetime used the electron-positron enhancement factor due to Barbiellini et al.^[2]^ described within the generalized gradient approximation. Positron lifetime calculations were performed for unrelaxed structure monovacancy defects and vacancy associates in Cu_7_S_4_ using 3 × 3 × 2 supercells.

**1.4. Measurements of enzyme-like activity**

1.4.1 POD-like

The POD-like activity of Cu_7_S_4_ nanozyme was elevated via the catalytic oxidation of the TMB with the assistance of H_2_O_2_ by spectrophotometrically monitoring the absorbance changes at 652 nm. Representatively, 20 µL of 15µg/mL Cu_7_S_4_ nanozymes solution (0.02M, pH 4.0 PBS), 20 µL of 10 mM TMB solution (0.02M, pH 4.0 PBS), 20 μL of 5 M H_2_O_2_ were added into 140 µL of 0.02M PBS (pH 4.0) solution. After 30 min, the catalytic oxidation of TMB was studied by the UV-Vis absorption spectra at 652 nm.

1.4.2 CAT-like

The generation of O_2_ was detected by the portable dissolved oxygen meter. 200 µL of 100 μg/mL Cu_7_S_4_ nanozymes solution, 800 µL of 5M H_2_O_2_ were added into 79 mL of 0.01M PBS (pH 7.2) aqueous solution under stirring and room-temperature conditions. Then, the content of dissolved oxygen was recorded every 30 s.

1.4.3 GSH-depletion

GSH-depletion was measured by the Ellman’s assay, and all the experiments were conducted in the dark and in triplicate.^[3]^ The Ellman reagent, 5,5'-dithiobis-(2-nitrobenzoic acid) (DTNB), is able to react with thiol groups (-SH) found within GSH. During this reaction, DTNB cleaves the disulfide bonds (-S-S-) of GSH, resulting in the formation of a yellow product (2-nitro-5-thiobenzoate acid). In a standard assay, 500 μL of 100 μg/mL Cu_7_S_4_ nanozyme (50 mM, pH 8.7, bicarbonate buffer solution) was incubated with 500 μL of 1.6 mM GSH (50 mM, pH 8.7, bicarbonate buffer solution) at room temperature for 1 h under stirring. 1 mM H_2_O_2_ + 0.8 mM GSH and 0.8 mM GSH solution were prepared as the positive and negative control groups, respectively. Afterward, these samples were centrifuged at 10000 rpm for 5 min. Then, 126 μL of Tris-HCl (50 mM, pH 8.0) solution and 2 μL of 25 mM DTNB (500 mM, pH 8.7, bicarbonate buffer solution) were added into 72 μL of supernatant. Finally, the absorbance at 412 nm was measured on a Microplate reader.

**1.5. Photothermal Performance of Cu_7_S_4_**

The aqueous Cu_7_S_4_ nanozymes solution and pure water were irradiated using an 808 nm laser (1.2 W/cm^2^) at room temperature for 600 s, respectively; the aqueous nanozymes solution (50 μg/mL) was irradiated for 600 s using different power densities (1.0, 1.2, and 1.4 w/cm^2^), respectively; and the laser with a power density of 1.2 w/cm^2^ was irradiated for 600 s using different concentrations (25, 50, 100 μg/mL) of the aqueous nanozymes solution for 600 seconds. Temperature changes and thermal images were captured with an infrared thermal camera (FLIR System E40). The aqueous solution of Cu_7_S_4_-1 nanozymes was irradiated at 1.2 W/cm^2^, and the temperature changes were recorded every 30 s to test the photothermal stability of the material (50 μg/mL) for four cycles.

**1.6. The density functional theory (DFT) calculations**

All periodic DFT calculations were carried out with the Vienna Ab Initio Simulation Package (VASP) code.^[4]^ The projector augmented wave (PAW) method,^[5]^ is used to describe the interactions between valence electrons and ion cores and the electronic wavefunctions are expanded in terms of a discrete plane-wave basis set. A plane wave energy cutoff of 400 eV was used. Electron exchange and correlation were treated with the generalized gradient approximation (GGA) within the Perdew-Burke-Ernzerhof (PBE) functional.^[6]^ Brillouin zone sampling was performed using a Monkhorst-Pack grid.^[7]^ The first Brillouin zone was sampled with a 1 x 1 x 1 k-point grid. All geometries were optimized using an energy-based conjugate gradient algorithm until the forces acting on each atom converged below 0.05 eV/Å. The vacuum height between slabs was 20 Å to eliminate the interaction between neighboring slabs. The Cu_7_S_4_ (224) is modeled by a supercell of 2 x 1 four-layer slabs. The two bottom layers were fixed to reduce the computational cost without influencing the accuracy of the simulations, while the remaining top layers and the adsorbates were allowed to fully relax.

**1.7. Bacteria culture**

The Luria-Bertani (LB) culture medium, normal saline (9 g of NaCl in 1000 mL of deionized water) and glassware were sterilized by autoclaving at 120 r for 30 min before the experiment. A monocolony of *E. coli* or *Pseudomonas aeruginosa* bacteria on a solid LB agar plate was transferred to 10 mL of LB liquid culture medium and cultured under shaking with 150 rpm at 37 °C overnight. The concentration of bacteria was estimated by the optical density (OD) value at the wavelength of 600 nm.

**1.8. Antibacterial activity of samples**

The inhibitory effect of the samples was determined by co-incubating the samples with bacteria and coating the plates to calculate the colony-forming units (CFU) and to plot the regeneration growth curves of the bacteria. In 50 mL glass conical flasks, 100 μL of the cultured bacteria was resuspended in 10 mL of normal saline. The Cu_7_S_4_ samples were then added into each conical flask to achieve the desired final concentrations. For the control group, normal saline was used instead of the Cu_7_S_4_ samples. The bacteria were incubated with two Cu_7_S_4_ samples at 37 °C for 1 h. For the CFU method, the bacteria solution was diluted 10, 100, 1000 or 10000 times with normal saline after incubation, and then 10 μL of the bacteria dilution was spread onto the LB agar plates. The number of CFU was recorded after incubation at 37 °C for 18 h. For the measurement of the bacterial regrowth curve, 5 mL of the bacteria solution was added into 10 mL of LB culture medium in 50 mL conical flasks, and then the mixture was cultured at 37 °C. Samples were taken at specific time intervals and the OD value at 600 nm was measured with a microplate reader. The bacterial regrowth curves were drawn by plotting the relationship between OD value and time.

For the photothermal antimicrobial assay, the bacteria were first incubated with two Cu_7_S_4_ samples at 37 °C for 30 min and then irradiated with an 808 nm laser (1.2 w/cm^2^, 10 min). After irradiation, the CFU counting method and the measurement of bacterial growth curves described above were used to detect the antibacterial efficiency of the samples.


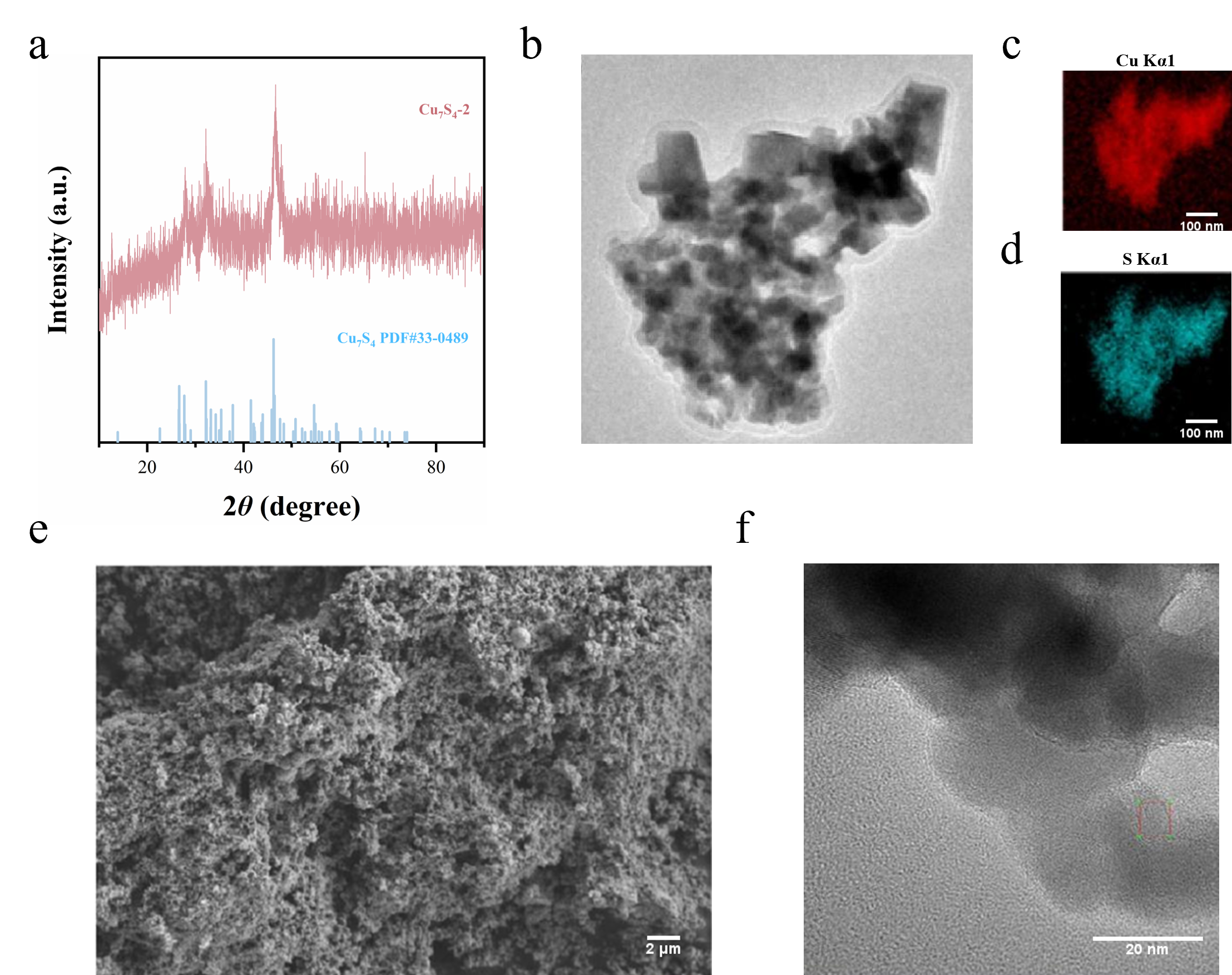


**Figure S1**. (a) XRD pattern of Cu_7_S_4_-2. b), (c), and (d) mapping images of Cu_7_S_4_-2, (c) show Cu element. (d) show S element, (e) SEM images of Cu_7_S_4_-2. (f) TEM image of Cu_7_S_4_-2.

**Table S1.** Positron Lifetime Parameters of Cu_7_S_4_

| Sample | τ_1_ (ps) | τ_2_ (ps) | τ_3_ (ps) | τ_4_ (ps) | I_1_(%) | I_2_(%) | I_3_(%) | I_4_(%) |
| --- | --- | --- | --- | --- | --- | --- | --- | --- |
| Cu_7_S_4_-1 | 221.6 | 355.0 | 1800.0 | 5800.0 | 29.1 | 67.5 | 3.6 | 0.37 |
| Cu_7_S_4_-2 | 226.0 | 374.0 | 1820.0 | 3500.0 | 34.5 | 59.6 | 5.2 | 0.7 |

**Table S2.** Calculated Positron Lifetime Values of Cu_7_S_4_

| Defect | Bulk | V*Cu* | V*CuS* | V*CuSCu* | *VCuCuCuSSS* | *VCuCuCuCuCuS* |
| --- | --- | --- | --- | --- | --- | --- |
| Lifetime (ps) | 187 | 223 | 276 | 289 | 355 | 374 |

**References**

1. J. M. C. Robles, E. Ogando, F. J. Plazaola, Positron lifetime calculation for the elements of the periodic table. *Phys: Condens, Mater*. **2007**, *19*, 176222.
2. B. Barbiellini, M. J. Puska, A. Harju, T. Torsti, R. M. Nieminen, Calculation of positron states and annihilation in solids: A density-gradient-correction scheme. *Phys. Rev. B*. **1996**, *53*, 16201.
3. W. Yin, J. Yu, F. Lv, L. Yan, L. R. Zheng, Z. Gu, Y. Zhao. Functionalized Nano-MoS2 with Peroxidase Catalytic and NearInfrared Photothermal Activities for Safe and Synergetic Wound Antibacterial Applications. *ACS Nano* **2016**, *10*, 11000-11011.
4. a) G. Kresse, J. Hafner. Ab initio molecular-dynamics simulation of the liquid-metal-amorphous-semiconductor transition in germanium. *Phys. Rev. B.*, **1994**, 49, 14251-14269; b) G. Kresse, J. Hafner. Efficiency of ab-initio total energy calculations for metals and semiconductors using a plane-wave basis set. *Comp. Mater. Sci.*, **1996**, 6, 15-50; c) G. Kresse, J. Furthmüller. Efficient iterative schemes for ab initio total-energy calculations using a plane-wave basis set. *Phys. Rev. B.*, **1996**, 54, 11169-11186.
5. a) G. Kresse, D. Joubert. From ultrasoft pseudopotentials to the projector augmented-wave method. *Phys. Rev. B.: Condens. Matter.* **1999**, 59, 1758-1775; b) P. E. Blöchl. Projector augmented-wave method. *Phys. Rev. B.*, **1994**, 50, 17953-17979.
6. J. P. Perdew, K. Burke, M. Ernzerhof. Generalized Gradient Approximation Made Simple. *Phys. Rev. Lett.* **1996**, 77, 3865.
7. H. J. Monkhorst, J. D. Pack. Special points for Brillouin-zone integrations. *Phys. Rev. B* **1976**, *13*, 5188-5192.
